# Can the Mindful Awareness and Resilience Skills for Adolescents (MARS-A) Program Be Provided Online? Voices from the Youth

**DOI:** 10.3390/children5090115

**Published:** 2018-08-28

**Authors:** Nicholas Chadi, Elli Weisbaum, Catherine Malboeuf-Hurtubise, Sara Ahola Kohut, Christine Viner, Miriam Kaufman, Jake Locke, Dzung X. Vo

**Affiliations:** 1 Department of Pediatrics, Boston Children’s Hospital, Harvard Medical School, 300 Longwood Avenue, Boston, MA 02115, USA; 2 Institute of Medical Sciences, University of Toronto, 1 King’s College Circle, Room 2374, Toronto, ON M5S 1A8, Canada; elliweisbaum@gmail.com; 3 Department of Psychology, Bishop’s University, 2600 College Street, Sherbrooke, QC J1M 1Z7, Canada; catherine.malboeuf.hurtubise@gmail.com; 4 Department of Psychiatry, Hospital for Sick Children, University of Toronto, 555 University Avenue, Toronto, ON M5G 1X8, Canada; sara.aholakohut@sickkids.ca; 5 Department of Pediatrics, Downstate Medical Center, State University of New York, 450 Clarkson Ave, Brooklyn, NY 11203, USA; christine.viner@gmail.com; 6 Division of Adolescent Medicine, Department of Pediatrics, Hospital for Sick Children, University of Toronto, 555 University Avenue, Toronto, ON M5G 1X8, Canada; meejum1@icloud.com; 7 Department of Child and Adolescent Psychiatry, British Columbia Children’s Hospital, University of British Columbia, Vancouver, BCV6H 3N1, Canada; jlocke@cw.bc.ca; 8 Division of Adolescent Health and Medicine, Department of Pediatrics, BC Children’s Hospital, University of British Columbia, 4480 Oak St, Vancouver, BC V6H 3N1, Canada; dvo@cw.bc.ca

**Keywords:** qualitative, mindfulness, meditation, chronic illness, adolescents, eHealth

## Abstract

Mindfulness-based interventions (MBIs) have been shown to improve health and well-being in adolescents with chronic illnesses. Because they are most often delivered in person in a group setting, there are several barriers that limit access to MBIs for youth with limited mobility or who cannot access in-person MBIs in their communities. The objective of this study was to determine if eHealth is a viable platform to increase accessibility to MBIs for teens with chronic illnesses. This study reports the qualitative results of a mixed method randomized trial describing the experience of the Mindful Awareness and Resilience Skills for Adolescents (MARS-A) program, an eight-week MBI, delivered either in person or via eHealth. Participants were adolescents between the ages of 13 and 18 with a chronic illness recruited at a tertiary pediatric hospital in Toronto, Canada. Individual semi-structured post-participation audio-video interviews were conducted by a research assistant. A multiple-pass inductive process was used to review interview transcripts and interpret emergent themes from the participants’ lived experiences. Fifteen participants (8 online and 7 in person) completed post-participation interviews. Four distinct themes emerged from participants in both groups: Creation of a safe space, fostering peer support and connection, integration of mindfulness skills into daily life, and improved well-being through the application of mindfulness. Direct quotations representative of those four themes are reported. Results from this study suggest that eHealth delivery of an adapted MBI for adolescents with chronic illnesses may be an acceptable and feasible mode of delivery for MBIs in this population. EHealth should be considered in future studies of MBIs for adolescents with chronic illnesses as a promising avenue to increase access to MBIs for youth who might not be able to access in-person programs.

## 1. Introduction

Adolescents with chronic illnesses face unique challenges that can have significant impacts on their development and well-being [1]. Adolescence is a period that typically involves the acquisition of independence and of a personal and social identity [2]. Managing a chronic illness and its associated appointments, procedures, and medications can disrupt this process [3]. Chronic health conditions (whether visible or not) can also cause emotional challenges and significant coping difficulties [4].

Mindfulness has been defined as “paying attention in a particular way: On purpose, in the present moment, and nonjudgmentally” [5]. Historically rooted in Eastern Buddhist and other contemplative traditions, mindfulness has recently gained popularity in both education and healthcare settings to promote health and well-being among adolescent populations [6]. In addition, mindfulness-based interventions (MBIs) for adolescents have been shown to have benefits on mood, stress, sleep, pain control, and concentration among many others in both clinical and non-clinical samples [7,8]. Emerging research has looked specifically at the feasibility and effectiveness of adapted MBIs for adolescents with chronic illnesses [9]. A randomized study conducted in 72 youth HIV-infected youth aged 14–22 who received an MBI or an active control intervention showed that mindfulness improved life satisfaction and cognitive accuracy in the context of negative emotion stimuli [10]. Another study conducted in 18 adolescents with functional somatic syndromes showed the high feasibility of an MBI and significant improvements in anxiety symptoms and level of functioning [11]. Finally, a number of small studies have shown that MBIs are well-received by adolescents with cancer and chronic pain conditions [12,13,14,15,16].

Despite these promising results, researchers have identified multiple barriers to implementation and dissemination of MBIs with adolescents with chronic illnesses. One barrier is that MBIs have traditionally been delivered in person in group settings [9]. This can pose a challenge for adolescents with mobility limitations, living in remote areas, or with limited access to transportation. Research conducted in adults has shown promise in the eHealth delivery of MBIs via online apps, web-based platforms, or hybrid modes of delivery [17,18,19,20]. To our knowledge, in-person and eHealth real-time group moderated MBIs have not been compared head-to-head in teens with chronic illnesses. Demonstrating the effectiveness of this new mode of delivery could have important implications to increase access to MBIs and potentially save costs of delivering this type of programming.

Studies conducted with adolescents have described the experience of participating in an MBI adapted for youth [21,22]. Qualitative data have revealed a number of benefits, including reduction of daily stressors and transformational shifts in life orientation and well-being [23]. It has been suggested that the incorporation of mindfulness practice in daily life demonstrates effective transmission of mindfulness skills to adolescents [24]. In this paper, we aim to describe the experience of adolescents with chronic illnesses receiving an MBI either in person or online. The overarching research question for this study was to determine if eHealth is a viable platform for teens with chronic illnesses. We hypothesized that eHealth would be an acceptable and feasible modality for the delivery of an adapted MBI in this population.

## 2. Materials and Methods

This paper will focus on the qualitative portion of a randomized mixed methods trial comparing the in-person and eHealth delivery of an adapted MBI for adolescents with chronic illnesses. All participants were included in both qualitative and quantitative analyses. Quantitative analyses sought to compare the acquisition of mindfulness skills and changes in mental health scores using standardized research questionnaires, changes in pre- and post-mindfulness salivary cortisol levels, and tracking of individual home practice between groups. The full description of the study protocol, including both qualitative and quantitative methods, has been published previously [25], and quantitative data will be reported separately. This study was a registered trial (ClinicalTrials.org: NCT03067207). All subjects gave their informed consent for inclusion before they participated in the study. The study was conducted in accordance with the Declaration of Helsinki, and the protocol was approved by the Ethics Committee of the Hospital for Sick Children in Toronto (project identification number: 1000053600).

Given our study’s mixed methods design and to allow more flexibility in interpretation of the data, we decided to use a generic qualitative approach, combining elements of qualitative description [26] and interpretive description [27], rather than being guided by an explicit set of philosophical assumptions from an established qualitative methodology [28,29]. For our analysis of the in-depth interviews, we were guided by an interpretive lens, allowing concepts to emerge from the lived experiences of the participants, rather than from our own preconceived notions [30].

Participants were adolescents aged between 13 and 18 recruited from different subspecialty clinics in a tertiary pediatric hospital in Toronto, Canada. Participants were eligible to participate if they lived close enough to attend weekly in-person sessions. They needed to have a diagnosis of a medical or mental health condition requiring ongoing medical care, which included at least one physical symptom, such as chronic pain or headaches. Potential participants were referred by one of their health providers and later contacted by a research assistant (CV) who invited them to a recruitment meeting where the details of the study were explained and written informed consent was obtained from the teen and parent/guardian. Interested participants were then randomized and allocated to an in-person or eHealth group. In total, 58 participants were assessed for eligibility, 40 were excluded for not meeting inclusion criteria, for declining to participate, or for other reasons, including living too far and only being interested in the eHealth arm of the study. Eighteen participants were randomized (9 per group) and 14 (7 in each group) completed the MBI and post-participation interview. Two participants who had not completed the MBI, but had attended at least two mindfulness sessions (both in the eHealth group), were invited to participate in a post-participation interview, and one of them accepted.

Both groups received an eight-week evidence-informed MBI, the Mindful Awareness and Resilience Skills for Adolescents [31] (MARS-A) program, delivered either via eHealth, through a secure online platform allowing audio-visual group interactions in real time (Zoom Video Conferencing—Zoom Video Communications Inc., San Jose, CA, USA), or in person at the hospital. MARS-A sessions are 90 min in length, and the curriculum and facilitation are adapted for adolescents from eight-week evidence-based adult MBI’s, including Mindfulness-Based Stress Reduction [32,33] and Mindfulness-Based Cognitive Therapy [34,35]. Each week, a different theme is explored, including introduction to mindfulness, informal mindfulness practice and gratitude, handling difficult emotions, and how best to take care of oneself. A more detailed thematic overview of the program can be found elsewhere [25,36]. Adaptations from adult MBIs include shorter sessions (90 min instead of 150 min), discussions focused on the challenges of coping with mental and physical illness in adolescence, and shorter group and home mindfulness practices. In-session activities included interactive group discussions related to weekly themes, mindfulness practices (i.e., sitting/breathing meditation, mindful movement, body scan, mindful eating, mindful listening), and review/inquiry of individual home practice. In-person and online groups were facilitated by co-authors, NC and EW, who both had extensive experience in MBIs for youth, were formally trained in teaching MARS-A, and had a long-standing personal mindfulness practice.

All participants took part in individual post-participation interviews through the same password-protected video conferencing platform used for the delivery of the MBI to the participants of the eHealth group. The video conferencing platform was chosen over phone or in-person interviews, since all participants, even participants in the in-person group, had been introduced to the platform at the time of enrollment. It was felt that this platform would allow a deeper connection with the interviewer than a phone interview and avoided the need for transportation for participants in the eHealth group. Interviews (average length 14 min, range 10–28 min) were conducted by a female research assistant with a medical degree (co-author, CV) who had received prior training in conducting semi-structured interviews and had met with participants at the moment of enrollment in the study. The interview template utilized was inspired by previous qualitative work with adolescents by co-authors, SAK [37] and NC [12]. Open-ended and semi-structured questions followed by specific probes were used to foster personal reflections about participants’ lived experience of the MARS-A program (see Appendix A). The interviews were approached as an opportunity for data to be co-created, with space given for participants to elaborate on each question. Participants completed the interviews from a quiet room at home and were made aware that all interviews were audio-recorded and de-identified prior to transcription and analysis.

Data was analyzed manually through a multiple-pass inductive process [38]. In a first pass, co-authors, CV, NC, and EW, independently read through all the transcripts and coded them to identify emergent themes. Codes were generated using participants’ own words whenever possible to minimize subjectivity. Co-authors, CV, NC, and EW, then met in person with the co-author, CMH, a child psychologist with extensive mindfulness facilitation experience who had reviewed the video-recordings of the MARS-A sessions to ensure facilitator quality and uniformity, and to review the themes that had been identified from the participants’ semi-structured interviews. From this analysis, four key themes emerged, and a common coding scheme was developed. In a second pass, transcripts were recoded by NC and EW using the common coding scheme. In a third pass, codes were compared by EW and placed into corresponding theme categories. A final review of the transcripts was conducted by NC and EW to discuss classification until consensus was reached and to ensure that the language of participants, and not that of the investigators, guided the theme names and descriptions.

## 3. Results

The average number of sessions attended among participants who completed the MBI was 6.7 in the in-person group and 7 in the eHealth group. The average participant age was 15.3 years. There were two male participants in each group and all four of them completed post-participation interviews. Participants who did not complete the intervention mentioned lack of time (online group) and difficulty with transportation (in-person group) as reasons for non-completion. Table 1 details participant characteristics at baseline.

Four prominent themes emerged from the interview data: (1) Creating a safe space; (2) fostering peer support and connection; (3) integration of mindfulness skills into daily life; and (4) improved well-being through the application of mindfulness. These themes were similar, with only subtle differences between the in-person and eHealth groups and reflected the lived experience of the MARS-A participants. For brevity and clarity, we have used ellipses, (…), to indicate pauses and square brackets, ([…]), to indicate edits/omitted material when reporting supporting excerpts from interviews [39].

### 3.1. Creating a Safe Space

During their interviews, participants in both groups used the word, “safe”, to describe their experience or environment. The use of the word, “safe”, or concept of “safe space” arose most frequently in relation to the question, “What did you like the most about the program?” For both in-person and eHealth participants, the non-judgmental attitude of other group members who were experiencing similar challenges related to their chronic health condition allowed them to feel at ease during the MBI.

- In-person participant (IPP): […] *it felt like a really safe space. Like it felt like you could really say whatever was on your mind*.
- IPP: […] *you could say what you need to say, so like, afterwards if you’re like stressed out about something then no one will like, judge you or make a comment…*
- EHealth participant (EHP): *I felt like it was a very safe environment… I felt like they were very welcoming, and it was comforting*.
- EHP: […] *they made you feel like whatever you were feeling was like OK […] Like, anytime I thought of something, I shared*.

### 3.2. Fostering Peer Support and Connection

Peer support and connection was reported in participants from both the in-person and online groups, with a focus on interaction with others either in person or through the audio-visual platform. These interactions were described as useful and allowed participants to compare their own challenges to those faced by their peers.

- IPP: *[…] you get to see like, other teens—what they’re going through and stuff [...] I thought it was really useful*.
- IPP: *[…] then we started to get to know each other and like before the sessions started, or like when it ended we would talk to each other afterwards*.
- EHP: *[…] it was really interesting to hear the other kids and what they were saying […] because I could relate to it, and I just didn’t expect other people to be going through the same thing as me…*
- EHP: *I liked how I was able to interact with other people… other people who are in similar situations as me*

### 3.3. Integration of Mindfulness Skills into Daily Life

Implementing mindfulness practices outside of the formal sessions was a theme that emerged from both groups. Participants in both groups reported several concrete examples of how they were able to use the skills learned during the MBI to help them in their everyday life.

- IPP: *I liked how we could always learn new practices every week. And I feel like being able to try the practices out at school really helped*.
- IPP: *I liked the eating [practice] one too, because I could fit that into my daily life*.
- EHP: *I’m working and in the process of forgiving a bunch of people in my life, […] so I definitely think that’s going to be a helpful thing*.
- EHP: *I liked the techniques that I learned, the different kind of mindfulness things we went through because I use them throughout the day all the time*.

### 3.4. Improved Well-Being through the Application of Mindfulness

Reports from participants in the in-person and online groups suggested a positive impact of the intervention on well-being. Some of the benefits mentioned included: A reduction of anxiety and mood symptoms, an improvement in sleep, and the acquisition of new coping skills.

- IPP: *I feel like I’m less anxious about stuff and I feel a little bit happier about stuff now*.
- IPP: *I know that a couple times my stress level was a little bit lower which was helpful […] like my sleeping patterns were consistent*.
- EHP: *I think the most useful part was how the program helps you cope with a lot of different things […] it just helps you cope all around*.
- EHP: *I don’t know what it is with the body scan, but it helps me go to sleep*.

## 4. Discussion

This article presents the qualitative results of a randomized study of an MBI for adolescents with chronic illnesses delivered in-person or via eHealth. The parallel emergence of four key themes—the creation of a safe space, the fostering of peer support and connections, the integration of mindfulness skills into daily life, and improved well-being through the application of mindfulness—in both the in-person and online groups suggests that an eHealth platform could present a viable alternative for the delivery of MBIs in this population.

Creating a safe space for learning, listening, and inquiry in which each participant feels comfortable and relaxed is an integral component of the MARS-A program and other eight-week mindfulness programs for adolescents [7,40]. This concept of creation of a safe space has been described in previous studies of adolescent MBIs [41] and was reported on several occasions by participants in both groups, yet, interestingly, was not included in any of the interview questions. Participants from the in-person group suggested that the lived experience of safe space allowed them to feel comfortable sharing their thoughts and emotions with the rest of the group. EHealth group participants’ reflections mirrored the experience of safe space described by the in-person group, also describing an ease to share with others. An important question that remains unanswered is whether the creation of a safe space was the result of a non-specific group effect (unrelated to the mindfulness intervention) or, rather, was related to the facilitators’ embodied mindfulness, which is considered to be integral to the pedagogy of mindfulness [42,43].

Although online participants’ interactions, unlike in-person participants, were limited to viewing one another on screen during the formal session, with no designated time for informal social interactions before, after, or during sessions, the theme of fostering peer support and connection was reported in both groups. There was a slight difference in the nature of the connection described by each group, with the word, “friendship”, used only by the in-person group and descriptors, like “similar”, used more frequently by the online group. Nevertheless, both groups reported benefiting from meeting others with whom they had a shared lived experience, which is frequently reported in in-person studies of MBIs in pediatric populations [11,16].

A primary research question was whether the feeling of support and mutual learning could be fostered within the confines of online interaction. Online facilitation comes with its own novel set of challenges [44]. One of these challenges is having less physical cues to the emotional state of participants. During in-person sessions, facilitators have the chance to observe participants in informal interactions before and after the session and during the break. These informal interactions are removed in the online setting, which did not appear to limit participants’ reports of group connectedness or the effectiveness of the intervention, as described in adult studies of MBIs delivered remotely [45].

One particularly striking finding regarding the group dynamic was seen in the reflection, “I got the sense that I wasn’t alone”, which was voiced by several participants. For individuals in the online group to feel this level of connection to their peers described in qualitative studies of in-person MBIs [15] speaks strongly to the viability of eHealth as a delivery platform for MBIs with adolescent populations. It also shows that a sense of community was fostered amongst participants, even though their description of their relationship to their peers—“friendship” vs. “support”—had a different quality between the in-person and online groups.

The question of whether the reported peer connectedness was attributed to the mindfulness intervention itself, or to a non-specific group effect, is challenging to answer without the presence of an active control group (e.g., non-mindfulness group therapy) [46,47]. It is worth noting that the intention of MBIs is usually not focused on fostering friendships per se, but rather on creating a sense of mindful peer connection and support [48], which was clearly reported by both groups. One could add that participants were part of a generation that has been exposed to technology and social media from an early age, which might have facilitated peer connection through the online platform [49].

Studies have indicated that pediatric participants in eight-week MBIs acquire the skillsets needed to apply mindfulness practices in their daily lives [11,15]. This integration of mindfulness skills can be assessed in different ways, including patient report and validated measurement scales [50], and is often used as a proxy to measure the effectiveness of MBIs [12,51]. In our study, participants were able to identify several real-life situations where the acquisition of new mindfulness skills would be useful for them (e.g., when eating, increased forgiveness). Both groups’ willingness and ability to apply diverse mindful practices outside of the sessions suggests a successful adoption of the practices via in-person and online modalities.

Pediatric in-person studies have shown that the application of mindfulness can improve participants’ well-being, specifically in relation to anxiety, pain, and sleep [14,16]. Both the in-person and online groups reported positive impacts in these three areas. In addition, participants explained that mindfulness had benefits on their happiness, body awareness, and reactions to difficult situations. On multiple occasions, participants went beyond simply describing foundational practices, such as sitting meditation, and were able to share high-level concepts, such as insight that their pain is connected to stress. This specific association has been described in previous in-person MBI studies, and is considered an indicator of increased mindfulness [16].

Qualitative data from post-participation interviews were meant to complement quantitative data from validated scales, logging of individual home practice, and measures of salivary cortisol levels also gathered during this study [52]. In brief, quantitative analyses from measurement scales and saliva samples were limited due to the small size of the sample, but there were similar levels of self-reported home practice between groups: In-person group: 6.5 times per participant/week (range = 1.4–13.4) and 28.8 min per participant/week (range = 4.3–154.7); and eHealth group: 6.0 times per participant/week (range = 2.9–9.7) and 30.6 min per participant/week (range = 6.6–107.8). Reported practices included all nine types of practices taught during MARS-A sessions (i.e., breathing meditation, body scan, mindful movement, eating meditation). These levels of individual practice, although much lower than those recommended (45 min per day) and observed (approximately 30 min per day) in adult MBIs [53], and lower than those reported in a recent in-person adolescent MBI study (median of 54 min per week) [11], provide some support that a number of different mindfulness practices were successfully integrated in participants’ everyday life.

None of the participants recruited for this study (including potential participants that ultimately were not enrolled in the study) identified access to the technology required to participate in the eHealth group (computer/tablet, web camera, microphone, internet connection) as a barrier to participation. While there were some technical issues that impacted individuals and the entire group, such as mild delays (3–5 min) in establishing a stable internet connection for all members at the beginning of five of the eight eHealth sessions, loss of connection for a 30 s to 8 min on a total of 12 occasions for four of the eHealth participants, and a defective microphone requiring reliance on instant messaging functions for one of the participants during two of the sessions, along with some distractions from their own space (e.g., family talking loudly or a pet coming into the room), most online participants reported that the online platform was convenient and easy to use and shared that being at home, versus commuting, resulted in more ease for them before, during, and after the session. Although this was not reported by study participants, studies of MBIs provided remotely suggest that learning mindfulness skills in one’s usual environment facilitates implementation of mindfulness skills and generalization to everyday life [54], with the added benefit of not having to plan additional time for transportation.

Study limitations: Due to a slower recruitment than anticipated, the sample size was small, and it is unclear if data saturation was reached. In addition, semi-structured interviews were short (less than thirty minutes), which could have limited the depth of participant reflections, and interviews took place immediately after the end of the MBI, which did not allow the capturing of any long-term effects of the intervention. In addition, interviews were conducted remotely through the same audio-video platform that was used for the delivery of the MBI in the eHealth group and was being trialed for feasibility. Finally, our study population was highly heterogeneous, limiting the possibility of applying findings to specific medical conditions. 

## 5. Conclusions

Qualitative results from this study suggest that eHealth may be an acceptable and feasible mode of delivery for MBIs for adolescents with chronic illnesses. Given the small size and preliminary nature of our study, more research is needed to confirm these findings. Nonetheless, eHealth should be considered as an alternative option in future studies of MBIs for adolescents, and as a promising avenue to increase access to MBIs for youth who might not be able to access in-person programs.

## Figures and Tables

**Table 1:** Participant characteristics at baseline.

Parameters	In-Person Group (*n* = 9)	Online Group (*n* = 9)
**Average age (range)**	15.2 (13–17)	15.4 (13–18)
**Gender (%)**		
Female	78	78
Male	22	22
**Ethnicity (%)**		
White/Caucasian	67	78
Asian	22	11
African-American	11	11
**Primary diagnosis (%)**		
Epilepsy	22	11
Anxiety	22	11
Somatic symptom disorder	11	11
Anorexia nervosa	11	11
Thalassemia	11	11
Diabetes	0	11
Obsessive-compulsive disorder	11	0
Juvenile idiopathic arthritis	0	11
Lupus	11	0
Cystic fibrosis	0	11
Asthma	0	11
**Chronic pain (%)**	44	44
**Sleep difficulties (%)**	67	78
**Mental health (%)**		
Any mental health diagnosis	89	78
Anxiety	78	67
Mood disorder	56	44
History of suicidal ideation	33	33
Other mental health diagnosis	33	22
**Currently taking medication (%)**	67	89
**Substance use (past month) (%)**		
Smoking/vaping (last month)	0	11
Alcohol (last month)	0	33
Other drugs	0	11
**Past yoga/tai chi experience (%)**	78	67
**Previous meditation/mindfulness experience**	44	44

## Appendix A

**Post-Intervention Semi-Structured Interview Guide**

	Participant ID: ________
Interviewer: ____________________________________________	
Date: __________________________________________________	
Start Time: _________ AM/PM End Time: __________ AM/PM	

Hello, my name is (insert research assistant’s name). I am one of the research assistants for the Mindfulness research project you are participating in at SickKids Hospital. First, I would like to thank you for participating in the research project. Now that you have completed the eight-week mindfulness program, I would like to ask you a few questions about the program. Our research team is interested in hearing your opinion about things that went well or were helpful for you and things that could be changed to improve the program in the future. This conversation should not take more than 15 to 20 min of your time. Is this still a good time for you to conduct this interview? [If no: Can I call you back at another time? What is the best time to reach you?]

1. **What did you like the most about the program?***Probe: What were some of the good things that came out of the mindfulness sessions?*
2. **What did you like the least about the program?***Probes: Were there some parts of the program that you didn’t like? Why? What ways could we improve on those parts?*
3. **What was the most useful part of the program for you?***Probes: Are there some things that you learned the program that you think you will use in the future?*
4. **What did you think of the teens in your group?***Probes: Did you enjoy being part of a group? If so, how or in what way? Was the size of the group too large/small? Did you find that you had enough/too much time to share your experience?*
5. (For participants in the eHealth arm) **What did you think of the iMeet platform?** *Probes: What issues, if any, did you have with the iMeet platform? What did you like/dislike about doing the program at home? Online/remotely?*
6. **How did you find your instructors?***Probes: Is there something that your instructors could have done differently? What did you like/dislike about your instructors?*
7. **Did you practice mindfulness between the sessions? If yes, approximately how many times a week did you practice and for how long? If no, why not?***Probe: What made it easy/difficult to practice at home? What were the barriers to home practice?*
8. (If yes at the previous question) **Which mindfulness practices did you do at home? Why did you choose this/these practice(s)?**
9. **What changes, if any, did you see in yourself after finishing the mindfulness program?***Probes: Better sleep, feeling happier, more energy, less anxious/stressed, less pain, increased school/social event attendance*
10. **Would you recommend the mindfulness program to a friend or to another young person with a health condition?***Probes: If no, why not? If yes, why would you recommend it?*
11. **Do you think that you will continue to practice mindfulness? Why or Why not?**
12. **Do you have any other comments that you would like to share with us about the program?***Probes: positive/negative experiences? General thoughts and feelings about program?*

Thanks again for your time and your participation in the program.
